# Combining EGFR-TKI With SAHA Overcomes EGFR-TKI-Acquired Resistance by Reducing the Protective Autophagy in Non-Small Cell Lung Cancer

**DOI:** 10.3389/fchem.2022.837987

**Published:** 2022-03-25

**Authors:** Peijun Cao, Yongwen Li, Ruifeng Shi, Yin Yuan, Hao Gong, Guangsheng Zhu, Zihe Zhang, Chen Chen, Hongbing Zhang, Minghui Liu, Zhenhua Pan, Hongyu Liu, Jun Chen

**Affiliations:** ^1^ Department of Lung Cancer Surgery, Tianjin Medical University General Hospital, Tianjin, China; ^2^ Tianjin Key Laboratory of Lung Cancer Metastasis and Tumor Microenvironment, Tianjin Lung Cancer Institute, Tianjin Medical University General Hospital, Tianjin, China

**Keywords:** EGFR-TKI, SAHA, autophagy, EZH2, EGFR-TKI acquired resistance

## Abstract

Nowadays, lung cancer has the highest mortality worldwide. The emergence of epidermal growth factor receptor (EGFR) tyrosine kinase inhibitors (TKIs) has greatly improved the survival of patients with non-small cell lung cancer (NSCLC) having EGFR-TKI-sensitive mutations. Unfortunately, acquired resistance happens for most patients. In the present research, we found that EGFR-TKIs (such as gefitinib and osimertinib) can induce autophagy in NSCLC cell lines. Compared with parental sensitive cells, drug-resistant cells have higher autophagy activity. The use of an autophagy inhibitor could enhance the toxicity of gefitinib and osimertinib, which indicates that the enhancement of protective autophagy might be one of the mechanisms of EGFR-TKI resistance in NSCLC. In addition, increased autophagy activity is associated with decreased enhancer of zeste homolog 2 (EZH2) expression. Knockdown of EZH2 or EZH2 inhibitor treatment could lead to increased autophagy in NSCLC cells, indicating that EZH2 is a negative regulator of autophagy. We revealed that the increase in autophagy caused by the reduction of EZH2 was reversed *in vitro* and *in vivo* when combining gefitinib or osimertinib with suberoylanilide hydroxamic acid (SAHA), a broad-spectrum histone deacetylase inhibitor (HDACi). In conclusion, our results indicated that the combination of EGFR-TKIs and SAHA may be a new strategy to overcome EGFR-TKIs acquired resistance.

## Introduction

Lung cancer as the leading cause of cancer-related death worldwide (Sung et al., 2021), and the most common type is non-small cell lung cancer (NSCLC) (Thai et al., 2021). The five-year survival rate for lung cancer patients is only approximately 15% due to the limited effectiveness of current conventional treatments, including surgery, radiotherapy, and chemotherapy, for patients with advanced lung cancer (Zhou et al., 2011; Miller and Hanna, 2021). In recent years, lung cancer treatment has entered the era of precise individualized treatment owing to the popularization of next-generation sequencing technology and the discovery of various oncogenic driver mutations (Zhou et al., 2011; Miller and Hanna, 2021). Among them, epidermal growth factor receptor (EGFR) tyrosine kinase inhibitors (TKIs) have become the first-line therapy for patients with advanced NSCLC harboring EGFR-TKI-sensitive mutations, considering their higher response rate and lower toxicity compared with conventional chemotherapy (Maemondo et al., 2010; Lee et al., 2017; Ramalingam et al., 2020). Unfortunately, despite the development of third-generation EGFR-TKIs, acquired drug resistance remains a difficult dilemma for EGFR-TKIs treatment (Remon et al., 2018). Studies have shown that there are various resistance mechanisms to different EGFR-TKIs, and approximately 20% of the resistance mechanisms are still not elucidated (Westover et al., 2018). Therefore, it is important to explore the possible resistance mechanisms to EGFR-TKIs and identify therapeutic options to reverse the acquired drug resistance.

Autophagy is a physiological mechanism that is commonly found in cells in normal and pathological states (Miller and Thorburn, 2021). In particular, under the conditions of intracellular and/or extracellular stress, such as hypoxia, nutrient deprivation, and pathogen infection, cells maintain cellular metabolic homeostasis by acquiring recyclable macromolecules such as nucleic acids and amino acids *via* autophagy; on the other hand, they can selectively remove certain cellular components, particularly damaged organelles, to maintain the stability of the intracellular environment. Therefore, autophagy may be considered as a potential mechanism to counteract cell killing (White et al., 2021). Autophagy has been described as a “double-edged sword” in the treatment of NSCLC with EGFR-TKIs. Wang et al. found that reduced autophagy was associated with resistance to erlotinib treatment (Wang et al., 2016a). Moreover, Li et al. showed that inhibition of osimertinib-induced autophagy enhanced the antitumor activity of osimertinib in lung adenocarcinoma (Li et al., 2019).

Enhancer of zeste homolog 2 (EZH2) belongs to the Polycomb group (PcG) family and plays an important role in epigenetic regulation as the main catalytically active subunit of Polycomb repressive complex 2 (PRC2) (Kim and Roberts, 2016). It has been shown that EZH2 exerts an essential role in the regulation of autophagy in tumor cells (Liu et al., 2020). Despite serving as a core member of the PRC2 complex, EZH2 expression is also regulated by protein translation modifications, and acetylation is one of the important modifications of EZH2 (Wan et al., 2015). A common finding in cancer cells is the high expression level of histone deacetylase (HDAC) isozymes and the corresponding hypoacetylation of histones, whereby HDAC is emerging as a prominent therapeutic target for cancer treatment (Libý et al., 2006; Nakagawa et al., 2007; Jenke et al., 2021). Suberoylanilide hydroxamic acid (SAHA), a broad-spectrum histone deacetylase inhibitor (HDACi), has also been shown to promote apoptosis induced by afatinib or third-generation TKI, including WZ4002 (Lee et al., 2015). However, the relationship between EZH2 acetylation modifications and autophagy is currently unknown.

In this study, we used gefitinib and osimertinib in combination with SAHA, respectively, and validated the inhibitory effect of this combination on tumor growth *in vitro* and *in vivo*. Moreover, SAHA was found to reduce gefitinib- and osimertinib-induced protective autophagy, and overcame acquired resistance to EGFR-TKIs.

### Cells Culture

NSCLC cell lines PC-9 and H1975 were obtained from the American Type Culture Collection. The PC-9/AB2 cell line was provided by Prof. Cai-Cun Zhou from Shanghai Pulmonary Hospital and the H1975OR cell line was provided by Prof. Jin-Jian Lu from the University of Macau (Ju et al., 2010; Tang et al., 2016). PC-9/AB2 and H1975OR were exposed to 1 μM gefitinib and 1 μM osimertinib, respectively, for a long period of time to maintain resistance. All cells were cultured in RPMI 1640 medium mixed with 10% fetal bovine serum, and were placed in a humid environment containing 5% CO_2_ at 37°C.

### Reagents and Antibodies

Gefitinib (ZD1839), osimertinib (AZD9291), SAHA, DZNep, and HCQ were purchased from Selleck Chemicals LCC. Antibodies against LC3A, p62, EZH2, mTOR, p-mTOR, TSC2, PCAF, and GAPDH were obtained from Cell Signaling Technology.

### Transfection and Drug Treatment

When the cells are in the logarithmic growth phase and cell fusion has reached approximately 80%, drug treatment and gene transfection can be performed. After discarding the old medium, DMSO (NC group) or drugs mixed with the medium were used to incubate the cells at working concentrations. Cells were transfected with siEZH2 (Ribobio, Guangzhou, China) and siNC using Invitrogen Lipofactamine 3000 according to the instructions. Treated cells were collected to obtain protein and RNA for further experiments 48 h later.

### Cell Counting Kit-8 Assay

Cell Counting Kit-8 (CCK-8) assay was performed as described in our previous publications (Gong et al., 2020). Briefly, 1 × 10^4^ cells were seeded in 96-well plates and incubated for 24 h. Cells were then treated with different concentrations of the drug for 48 h. Next, the CCK-8 reagent and medium were added to each well at a ratio of 1:10, followed by incubation at 37°C for 1 h. After incubation, absorbance was measured at 450 nm using a microplate reader (SpectraMax M5, Molecular Devices, Sunnyvale, CA, United States). The experiment was repeated at least three times.

### Colony Formation Assay

Approximately 500 cells in the logarithmic growth phase were inoculated in each well of a 6-well plate and incubated at 37°C for 24 h. The cells were then treated with the working concentrations of the drugs. After roughly 14 days, the colonies were fixed with methanol and stained with 0.5% crystal violet for 30 min at room temperature. The number of colonies (defined as >50 cells) was counted and photographed.

### Flow Cytometry Analysis of Apoptosis

Apoptotic cells were detected using membrane-linked protein V-FITC and PI staining according to BD’s Apoptosis Detection Kit instructions. The drug-treated cells for the assay were washed, collected, and transferred to flow cytometry tubes after warming with 500 ml of binding buffer. Then, 5 μl of Annexin V-FITC and 5 μl of PI were added, followed by incubation for 15 min at room temperature in dark condition. The stained cells were analyzed by NovoCyte flow cytometry (Agilent, United States).

### Western Blotting

The experiments were performed according to the method described in our published article (Zhang et al., 2021). A summary of the procedure is described as follows: After extraction of the cellular proteins, the protein concentration was measured by BCA assay (Thermo Fisher Scientific, Inc., Waltham, MA, United States), ensuring that the total amount of protein in each group of samples was 30 μg. After 2 h at a constant current of 250 mA (time can be adjusted appropriately according to the molecular weight of the target protein) the proteins in the gel were transferred to a PVDF membrane (Millipore, Billerica, MA, United States). The membranes were then blocked with 5% skim milk at room temperature for 2 h. The primary antibody and membranes were incubated overnight at 4°C. Subsequently, the membranes and secondary antibody (1:5000 dilution; Thermo Fisher Scientific, Inc.) were incubated for 1 h at room temperature. The bands were visualized using the Pierce ECL substrate (Thermo Fisher Scientific, Inc.).

### Coimmunoprecipitation

To determine the cell concentration of each sample, trypsin-digested cells were precipitated, washed with PBS, resuspended, and counted using a cell counter (Bio-Rad TC20, CA, United States). Different volumes of RIPA lysis buffer were added to ensure the same total cell count for each sample according to the cell concentration of each sample. The samples were then incubated with rabbit anti-acetylated-lysine antibody (Cell Signaling Technology, Inc., MA, United States) or normal rabbit IgG antibody (Beyotime, Shanghai, China) at 4°C 24 h. The next day, protein A/G agarose beads (Beyotime, Shanghai, China) were added to the samples and slowly mixed for 3 h at 4°C in a refrigerator, then samples were centrifuged to remove the supernatant and the beads were washed five times with lysis buffer. After centrifugation, the precipitate was dissolved in SDS loading buffer and boiled at 100°C for 10 min. Protein blotting was performed with rabbit anti-EZH2 antibody.

### Autophagic Flux Measurement

Cells were cultured on slides, transfected with GFP-RFP-LC3 adenovirus (HanBio, Shanghai, China), and then treated with different small molecule compounds for 48 h. The cells were fixed in 4% paraformaldehyde after washing with PBS buffer. The slides were then blocked with DAPI-containing anti-quenching agent, and the localization of LC3 spots was observed by confocal fluorescence microscopy. The cell nuclei were stained with DAPI. Fluorescence images were captured using a confocal microscope (ZEISS, Germany) to detect autophagosomes (yellow dots in fusion images) and autolysosomes (red dots in fusion images).

### RNA Extraction and Quantitative PCR Assays

Total cellular RNA was extracted by Trizol (Invitrogen, CA, United States), quantified by a UV spectrophotometer (Beckman Coulter, CA, United States), and 1 μg of total RNA was reverse-transcribed by a PrimeScript RT kit (TaKaRa, Dalian, China). The obtained cDNA was mixed with ABI (ABI, CA, United States) SYBR Green Master Mix and the corresponding gene primers and subsequently amplified on an ABI 7900 real-time quantitative PCR instrument. The expression level of EZH2 was normalized to the expression level of the glyceraldehyde-3-phosphate dehydrogenase (GAPDH) –ΔΔCT method. All gene primers were obtained from BGI (Guangdong, China).

### Immunohistochemistry

Mouse xenograft tumor tissue was fixed overnight with 4% formalin, dehydrated using ethanol, and embedded in paraffin. Next, the paraffin blocks were cut into 5-μm-thick slices. The prepared tissue slices were first deparaffinized in xylene and then rehydrated with a gradient alcohol solution. After blocking with normal goat serum for 10 min, the sections were incubated with EZH2-specific antibody (1:50 dilution) and LC3A-specific antibody (1:1000 dilution) for 1 h at room temperature, washed with PBS, and incubated with secondary antibody (ZSJQ, Beijing, China) for 60 min. Finally, the cells were incubated with 3,3′-diaminobenzidine for 3 min at room temperature and counterstained with hematoxylin.

### *In vivo* Study

The source of nude mice and the feeding environment were consistent with those described in our previous publication (Gong et al., 2020). PC-9/AB2 cells (2 × 10^6^) were injected subcutaneously into the left groin of nude mice. When the tumor volume reached approximately 200 mm^3^, mice were randomly divided into four groups (five mice/group). Drugs were prepared with sodium carboxymethylcellulose (CMC-Na). Tumor size was measured every other day in the NC group (CMC-Na), Gef group (50 mg/kg/day), SAHA group (50 mg/kg/day), and Gef + SAHA group (50 mg/kg/day of gefitinib plus 50 mg/kg/day of SAHA). Tumor volume (V) was calculated by the following formula: volume (mm^3^) = [length × width^2^ (Thai et al., 2021)] × 0.5. After 4 weeks, tumors were isolated from mice and stored in paraformaldehyde at 4°C. The experiments on the animals were approved by the ethics committee of Tianjin Medical University General Hospital.

### Statistical Analysis

Statistical analysis was performed by GraphPad Prism8 (GraphPad Software Inc., CA, United States). Differences between groups were analyzed by one-way ANOVA followed by Dunnett’s *t*-test for individual comparisons. When the comparison involved only two groups, the Student’s *t*-test was used. Tumor sizes in the nude mice and results of the CCK-8 assay were analyzed for differences using two-way ANOVA. *p* < 0.05 was determined to represent a significant difference.

## Results

### EGFR-TKIs Induced Protective Autophagy in NSCLC Cells

**FIGURE 1:**
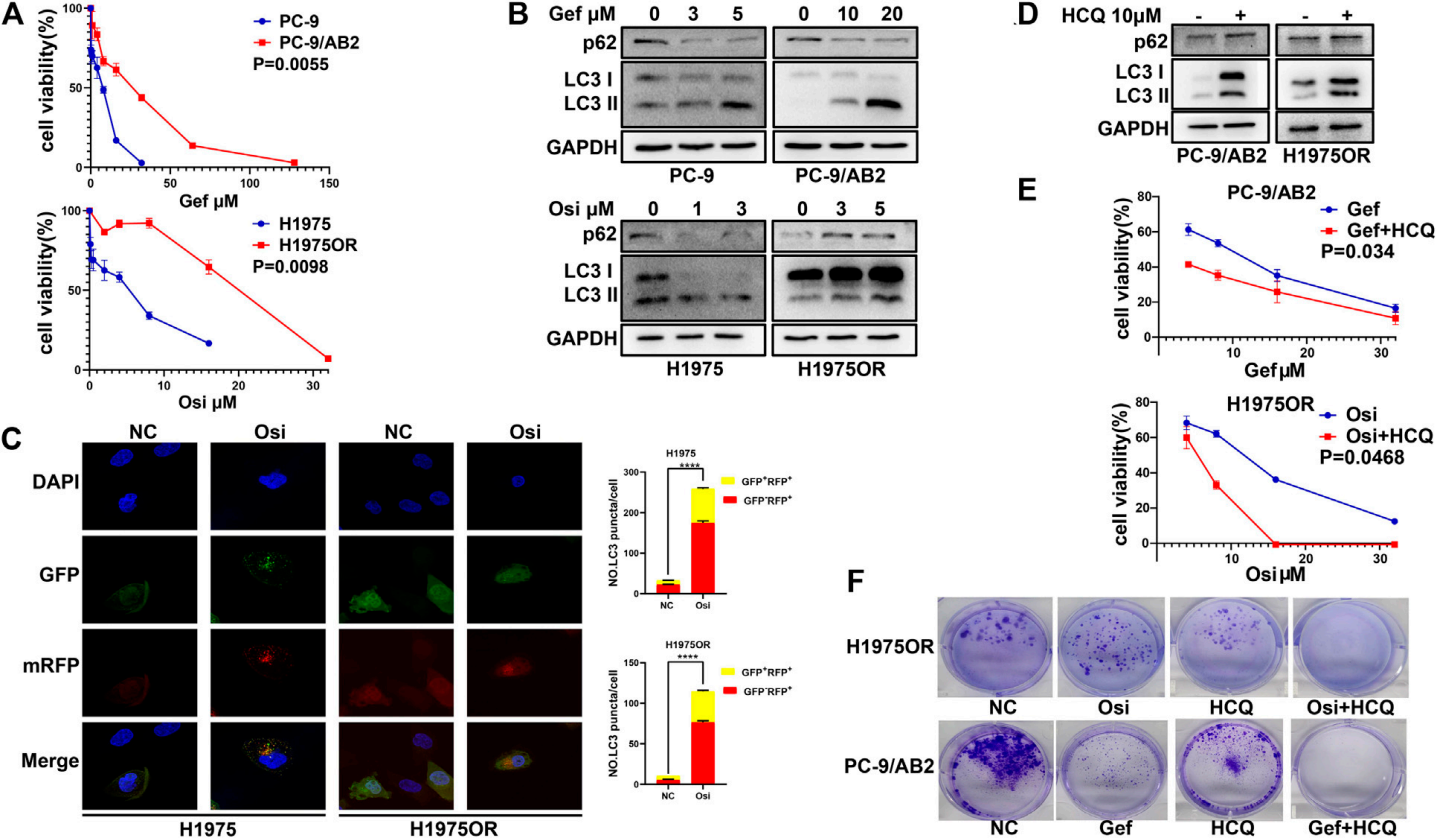
EGFR-TKIs induced protective autophagy in NSCLC cells. **(A)** The CCK-8 method was used to detect parental PC-9 and H1975 cells and their corresponding drug-resistant cells, and the concentration of gefitinib or osimertinib was increased and treated for 48 h. Three replicates were used for the experiments, and data are presented as mean ± SEM. **(B)** EGFR-TKIs treatment decreased p62 protein levels and increased LC3-II accumulation in PC-9/H1975 and corresponding drug-resistant cells. **(C)** Fluorescence assay showing representative images of cells transfected with GFP-RFP-LC3 double-labeled adenovirus. Autophagic flux was significantly increased in H1975 and H1975OR after 1 μM osimertinib treatment for 48 h. Quantitative analysis of the number of yellow autophagosomes and red autolysosomes. ****p < 0.0001. **(D)** 10 μM HCQ can inhibit the autophagy level of drug-resistant cells. **(E)** CCK-8 assay suggested that the inhibitory effect on drug-resistant cells was significantly enhanced after EGFR-TKIs combined with 10 μM HCQ compared with single EGFR-TKI treatment. Three replicates were used for the experiments, and the data are presented as mean ± SEM. **(F)** The colony formation assay suggested that the number of colonies in the two-drug combination group was significantly lower than that in the single-drug group. NC: DMSO, Gef: 1 μM gefitinib, Osi: 1 μM osimertinib, HCQ: 10 μM HCQ, Gef + HCQ: 1 μM gefitinib + 10 μM HCQ, Osi + HCQ: 1 μM osimertinib + 10 μM HCQ.

Firstly, the IC50 of the gefitinib-sensitive cell line PC-9 with its corresponding drug-resistant cell line PC-9/AB2, and the osimertinib-sensitive cell line H1975 with its corresponding drug-resistant cell line H1975OR were investigated by CCK-8 assay (Figure 1A). To explore the effects of EGFR-TKIs on autophagy in NSCLC cells, the expression of p62 and LC3 were observed by western blotting (Figure 1B) or based on GFP^+^RFP^+^ and GFP^−^RFP^+^ puncta in cells using confocal microscopy (Figure 1C). The results displayed that both sensitive and drug-resistant cells treated with EGFR-TKIs showed increased LC3-II accumulation and decreased p62 expression. A significant increase of GFP^+^RFP^+^ yellow puncta representing the autophagosome and GFP^−^RFP^+^ red puncta representing the autolysosome could be seen in the cytoplasm under confocal microscopy (Yu et al., 2017). Which suggests that autophagy is prevalent in NSCLC cells treated with EGFR-TKIs. The completion of autophagy ultimately relies on the autolysosome to perform degradation functions. Hydroxychloroquine (HCQ) can inhibit autophagy by elevating the pH of the lysosome, inhibiting the function of the lysosome, and preventing the degradation of LC3-II (Wang et al., 2016b). After treating drug-resistant cells with 10 μM HCQ, we found that HCQ inhibited the degradation of p62 and increased the accumulation of LC3-II (Figure 1D). When combining treatment of EGFR-TKIs and HCQ in drug-resistant cells, both the CCK-8 and colony formation assays showed that HCQ enhanced the antitumor activity of EGFR-TKIs in drug-resistant cells (Figures 1E,F). These results suggested that the autophagy induced by EGFR-TKIs was protective in NSCLC cells.

### EZH2 is a Negative Regulator of Autophagy

**FIGURE 2:**
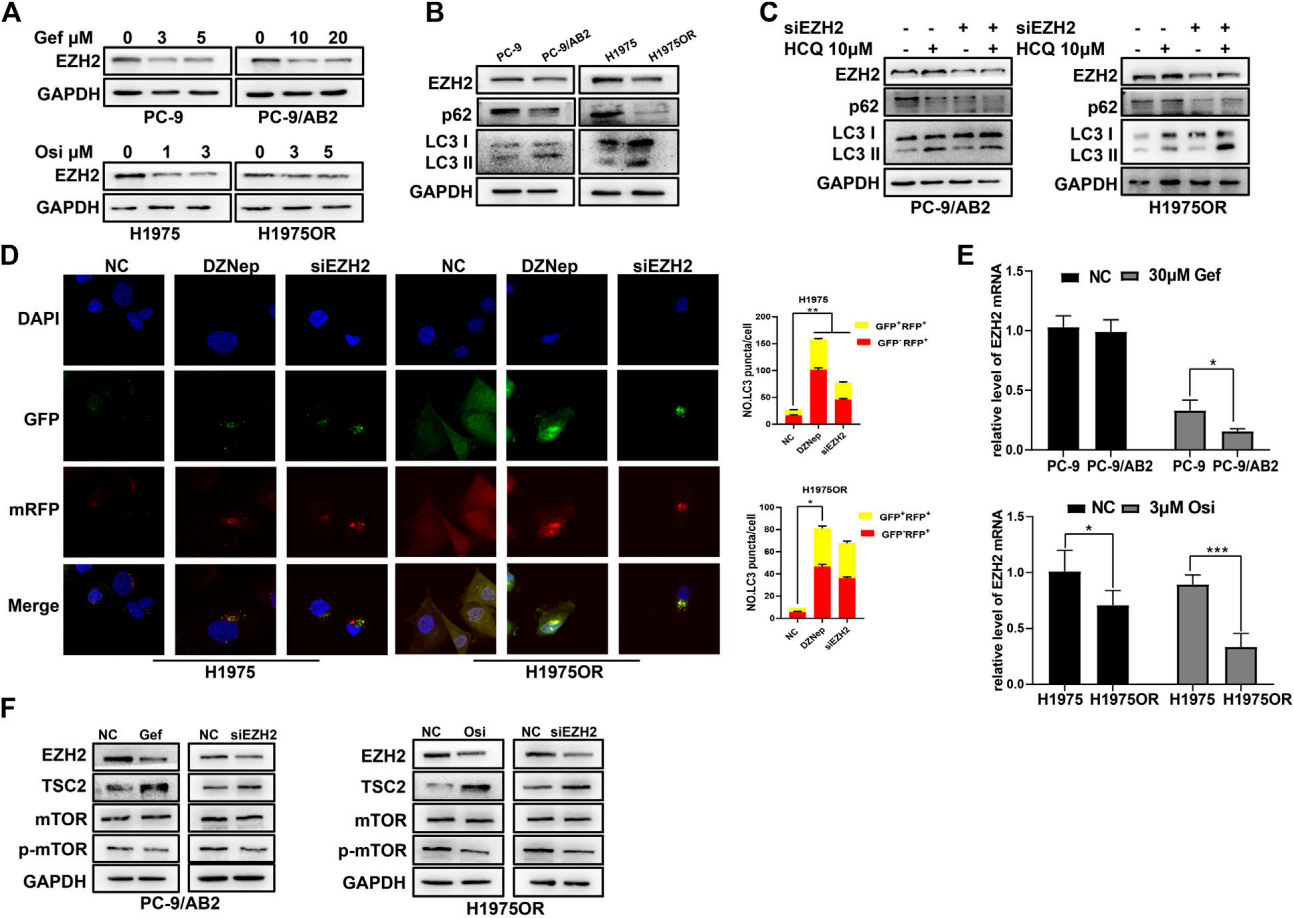
EZH2 is a negative regulator of autophagy. **(A)** EGFR-TKIs can cause decreased expression of EZH2 protein in PC-9/H1975 and corresponding drug-resistant cells. **(B)** Western blotting revealed lower levels of EZH2 protein and higher autophagic activation in drug-resistant cells. **(C)** Western blotting showed enhanced autophagic activity of drug-resistant cells following transfection with siEZH2. **(D)** Fluorescence assay showing representative images of cells transfected with GFP-RFP-LC3 double-labeled adenovirus. Autophagic flux was increased after knockdown of EZH2 in H1975 and H1975OR. Quantitative analysis of the number of yellow autophagosomes and red autolysosomes. *p < 0.05, **p < 0.01. **(E)** RT-qPCR assays revealed lower levels of EZH2 mRNA in drug-resistant cells. **(F)** Western blotting showed that downregulation of EZH2 induced by EGFR-TKIs affects the mTOR signaling pathway and its suppressor TSC2.

When treated with different concentration gradients of EGFR-TKIs, expression of EZH2 was found to be declining in NSCLC cells (Figure 2A). The expression of EZH2 was lower in drug-resistant cells compared to sensitive cells, and the decreasing of p62 expression and the increasing of LC3-II accumulation in drug-resistant cells implied a higher degree of autophagy (Figures 2B,E). To explore the relationship between EZH2 and autophagy, EZH2 inhibitor DZNep and siRNA were employed to knock down EZH2 expression. Only the decreasing of p62 was observed when treated with DZNep or siRNA alone in drug-resistant cells (Supplementary Figures S1A,B). Considering that cellular autophagy is a dynamic process, the degradation of LC3-II also increases when the degree of autophagy is enhanced. When DZNep/siEZH2 and HCQ were combined in drug-resistant cells, the degradation of LC3-II was found to be repressed, and p62 expression was decreased (Figure 2C, Supplementary Figure S1C). Similarly based on the findings of confocal microscopy, an increase in autophagosomes and autolysosomes was observed in the cells in which EZH2 expression was inhibited (Figure 2D). These results suggest that drug-resistant cell autophagy is significantly increased after EZH2 inhibition, and that EZH2 is a negative regulator of cellular autophagy activation.

It was recently shown that EGFR activation induces the activation of the mammalian target of rapamycin complex 1 (mTORC1) pathway, which in turn inhibits the formation of autophagosomes (Wei et al., 2015; Kwon et al., 2019). In our study, the mTOR signaling pathway was downregulated when treated with EGFR-TKIs or transfected with siEZH2 in drug-resistant cells, which was accompanied by an increase of TSC2, an mTOR signaling pathway inhibitor (Figure 2F). It indicates that downregulation of EZH2 plays a large role in autophagy induced by EGFR-TKIs in NSCLC cells.

### SAHA Reversed the Effects of Increased Autophagy and Decreased EZH2 Expression Induced by EGFR-TKIs

**FIGURE 3:**
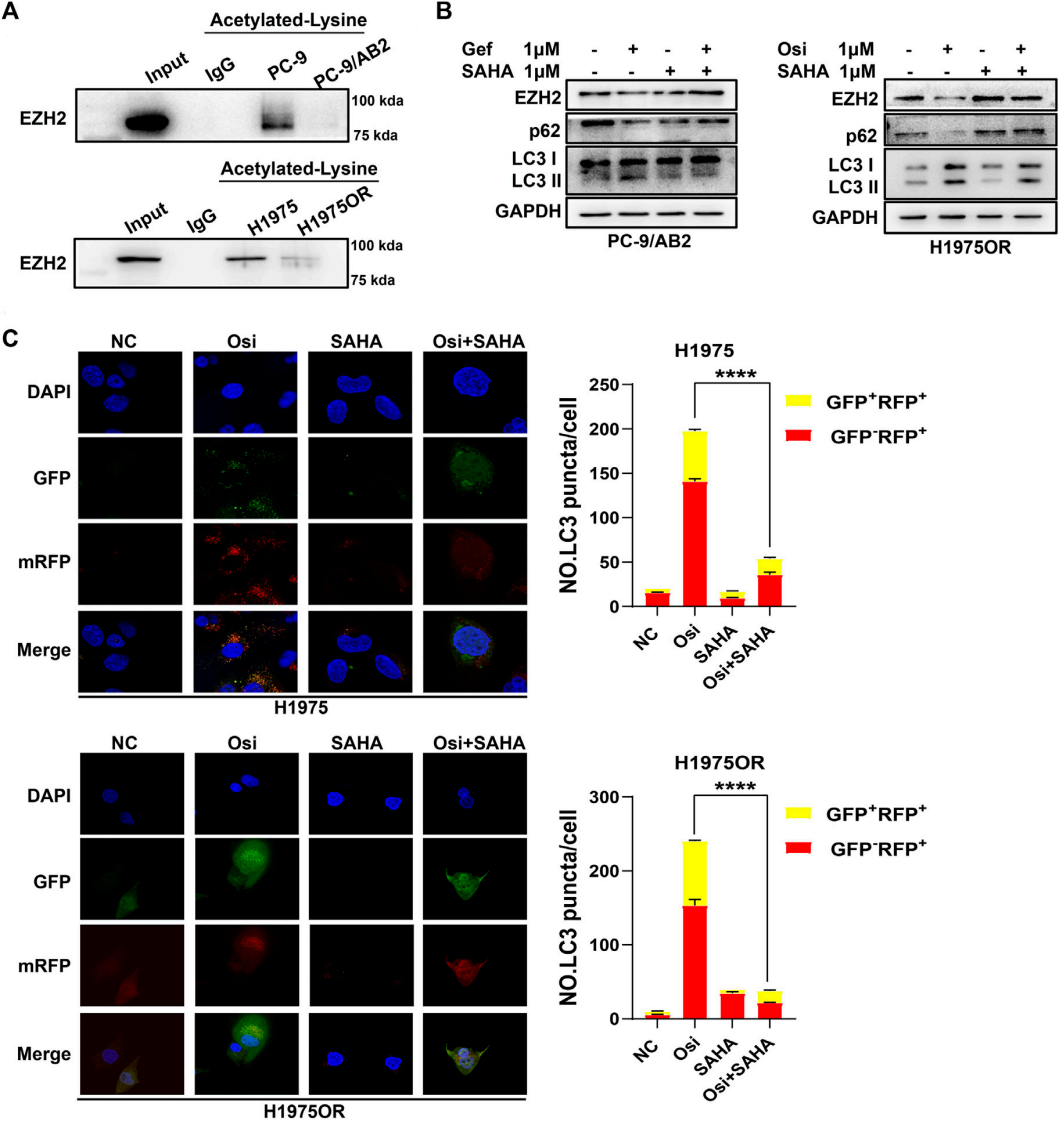
SAHA reversed the effects of increased autophagy and decreased EZH2 expression induced by EGFR-TKIs. **(A)** The acetylation level of drug-resistant cells was lower than the corresponding sensitive cells. After treatment of cells with 1 μM SAHA for 48 h, cell lysates were immunoprecipitated with rabbit anti-acetylated-lysine antibody or normal rabbit IgG antibody, followed by anti-EZH2 antibody for immunoblotting. **(B)** Western blotting showed that SAHA reversed the effects of EGFR-TKIs on EZH2 protein expression and autophagy in drug-resistant cells. **(C)** Fluorescence assay showing representative images of cells transfected with GFP-RFP-LC3 double-labeled adenovirus. SAHA reversed the increased autophagic flux induced by EGFR-TKIs. ****p < 0.0001.

Existing studies have shown the presence of EZH2 acetylation modifications in NSCLC cells (Wan et al., 2015), but acetylated EZH2 is difficult to detect *via* western blotting after immunoprecipitation with rabbit anti-acetylated-lysine antibody, if cells are not treated with any deacetylase inhibitors. We treated the cells with SAHA a broad-spectrum deacetylase inhibitor and then detected the acetylated modification of EZH2 in the cells by co-immunoprecipitation. The results of the coimmunoprecipitation assay showed that the level of EZH2 acetylation was lower in drug-resistant cells compared to parental cells (Figure 3A). To further explore the effect of SAHA on EZH2 expression and autophagy, we treated the drug-resistant cells with EGFR-TKI in the presence and absence of SAHA. It showed that no changes of EZH2 and autophagy were found in drug-resistant cells when treated with SAHA alone (Supplementary Figure S1D). The downregulation of EZH2 and increased autophagy activation was found to be induced by EGFR-TKIs, while SAHA reversed these effects when combined with EGFR-TKIs (Figures 3B,C). These data confirm that SAHA can reverse the reduced EZH2 expression and increased autophagy caused by EGFR-TKIs.

### SAHA Enhanced the Antitumor Effect of EGFR-TKIs *in vitro*

**FIGURE 4:**
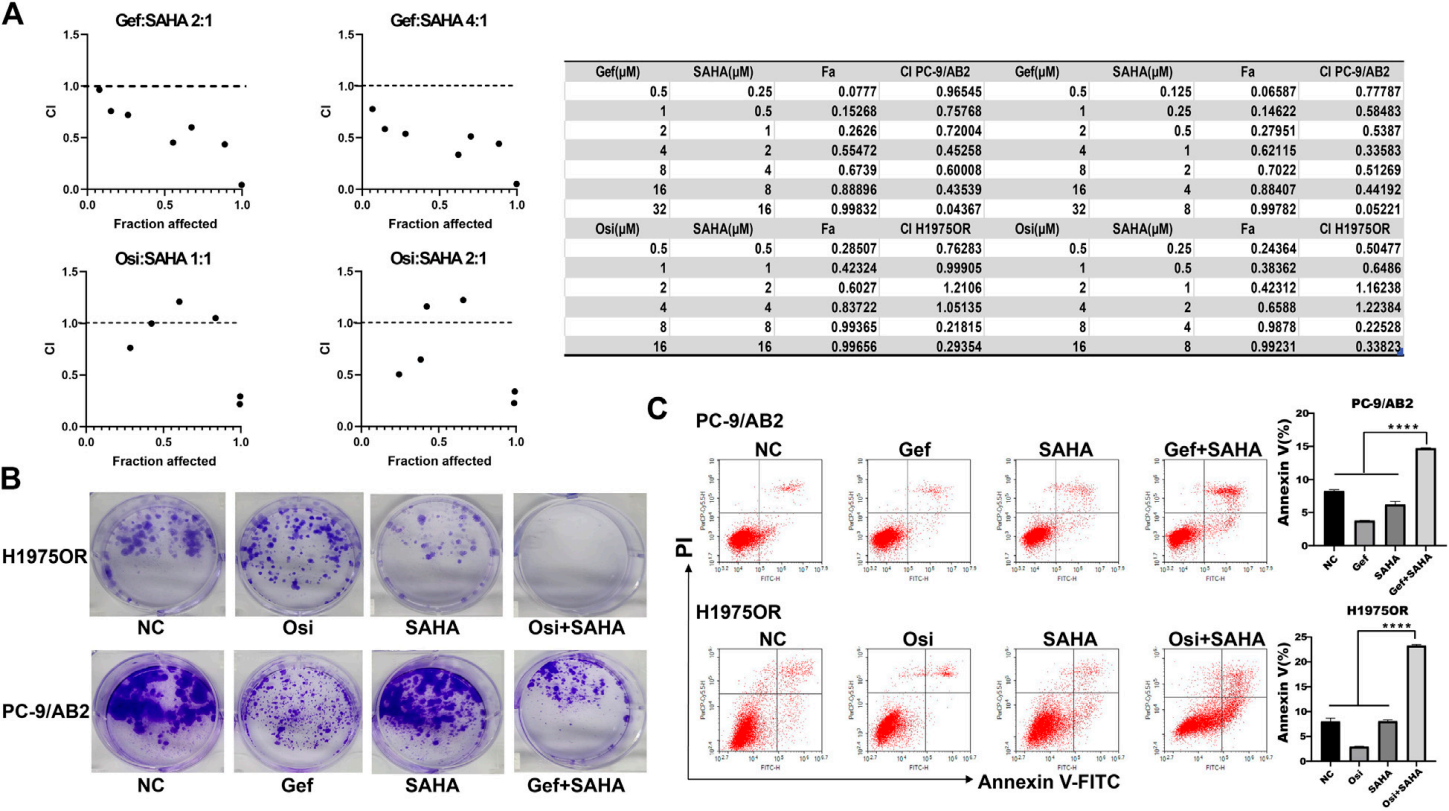
SAHA enhanced the antitumor effect of EGFR-TKIs *in vitro*. **(A)** PC-9/AB2 and H1975OR cells were incubated with different concentrations of gefitinib or osimertinib combined with SAHA for 48 h. Cell viability was determined using the CCK-8 method. The combination effect of the two drugs was then evaluated according to the combination index (CI). **(B–C)** NC: DMSO, Gef: 1 μM gefitinib, Osi: 1 μM osimertinib, SAHA: 1 μM SAHA, Gef + SAHA: 1 μM gefitinib + 1 μM SAHA, Osi + SAHA: 1 μM osimertinib + 1 μM SAHA. The colony formation assay suggested that the number of colonies in the two-drug combination group was significantly lower than that in the single-drug group. Detection of apoptosis by flow cytometry revealed that the proportion of apoptotic cells in the two-drug combination group was much higher than that in the single-drug and NC groups. The bar graph shows the percentage of apoptotic cells in different groups (*n* = 3, ****p < 0.0001).

In light of the anti-autophagic effects of SAHA *in vitro*, to further determine whether SAHA enhanced the antitumor effects of EGFR-TKI in drug-resistant cells of NSCLC, we investigated the effects of the combination of EGFR-TKIs and SAHA on the viability of PC-9/AB2 and H1975OR using the CCK-8 assay. For PC-9/AB2, we used a combination of gefitinib and SAHA at fixed concentration ratios of 2:1 and 4:1, and for H1975OR, we used a combination of osimertinib and SAHA at fixed concentration ratios of 2:1 and 1:1. The combination index was calculated by the CompuSyn software (Hsu et al., 2020). A combination index (CI) less than 1 means that the combined drugs have a synergistic effect. Generally, when considering the therapeutic effects of antitumor drugs, CI values, which correspond to high inhibition rates, are given more importance. In our study, the CIs of Gef + SAHA (2:1/4:1) and Osi + SAHA (1:1/2:1) were 0.19228/0.19353 and 0.45807/0.44211, respectively, at a 97% inhibition rate, as calculated by the mathematical model proposed by Chou-Talalay, which suggested that the co-administration of SAHA with EGFR-TKIs had a stronger antitumor effect than administration of EGFR-TKI alone (Figure 4A). Similar results were obtained by the colony formation assay (Figure 4B). In addition, the proportion of apoptotic cells was significantly increased in EGFR-TKIs-resistant cells when administered SAHA and EGFR-TKIs (Figure 4C). These results showed that SAHA can enhance the antitumor effects of EGFR-TKIs on drug-resistant cells *in vitro*.

### SAHA Enhanced the Antitumor Effect of EGFR-TKIs *in vivo*

**FIGURE 5:**
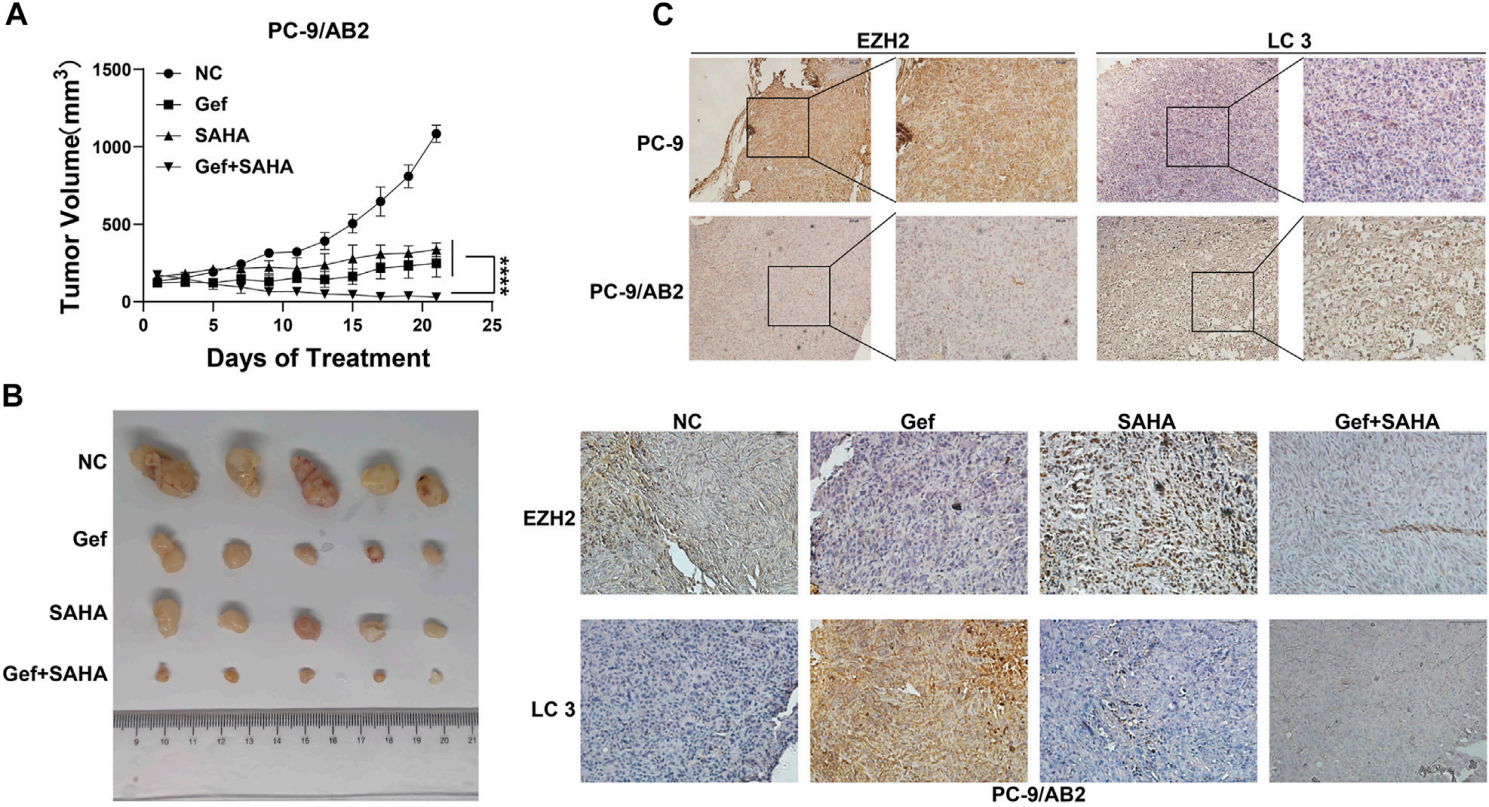
SAHA enhanced the antitumor effect of EGFR-TKIs *in vivo*. **(A–B)** Nude mice with tumors of PC-9/AB2 cells received gefitinib (50 mg/kg orally), SAHA (50 mg/kg orally), and a Gef + SAHA combination after 3 weeks of treatment every other day. Results are presented as mean ± standard deviation. ****p < 0.0001. **(C)** Immunohistochemistry was performed to detect EZH2 and LC3 in tumor sections of nude mice.

To validate the antitumor effects of the co-administration of SAHA with EGFR-TKI *in vitro*, we established *in vivo* xenografts in BALB/c nude mice using PC-9/AB2 cells. When tumors were palpable, mice were intragastrically administered gefitinib (50 mg/kg), SAHA (50 mg/kg), or the combination of both drugs for 3 weeks, respectively. As shown in Figures 5A,B, gefitinib and SAHA monotherapy led to only a slight reduction in tumor volume, while co-administration of SAHA with EGFR-TKI resulted in significant tumor shrinkage (Figures 5A,B). To further investigate the mechanism underlying the antitumor effect, we analyzed xenograft tumor sections using immunohistochemistry to verify EZH2 and LC3 expression. Immunohistochemical analysis showed that lower EZH2 expression and higher LC3 expression were found in the resistant tumor tissues compared to parental sensitive cells, and the combined treatment of SAHA with gefitinib could reverse the decrease in EZH2 and increase in LC3 caused by gefitinib, which was consistent with the *in vitro* results (Figure 5C, Supplementary Figure S2B). To conclude, our results confirmed that SAHA enhances the antitumor effect of EGFR-TKIs in EGFR-TKI-resistant *in vivo* xenografts.

## Discussion

In clinical practice, treatment targeting specific oncogenic driver mutations can inhibit tumor progression and prolong patient survival (Yang et al., 2020). Epidermal growth factor receptor (EGFR) mutations activated in non-small cell lung cancer (NSCLC) are effective targets for EGFR tyrosine kinase inhibitors (TKIs) (Zhou et al., 2011). Patients with EGFR mutation-positive lung adenocarcinoma have approximately an 80% response rate to EGFR-TKIs, achieving progression-free survival of 10–14 months (Wu and Shih, 2018). Unfortunately, the emergence of acquired resistance to EGFR-TKIs has limited the long-term use of this therapy (Wu and Shih, 2018; Terlizzi et al., 2019). Current studies have found that the most common mechanism of resistance with first- or second-generation EGFR-TKIs is the development of acquired EGFR T790M mutations, accounting for approximately 50% of cases (Tan et al., 2018). For this reason, third-generation EGFR-TKIs, represented by osimertinib, have emerged to treat patients with acquired resistance mutations in T790M. However, third-generation EGFR-TKIs still cannot prevent the development of acquired resistance due to persistent mutations in the gene (Lee, 2017; Remon et al., 2018). An increasing number of studies have shown that combining existing small molecule anticancer drugs with EGFR-TKIs can circumvent acquired drug resistance and enhance the antitumor effects of EGFR-TKIs through a bypass signaling mechanism (Rosell et al., 2021). In the present study, we found that the combination of EGFR-TKIs and SAHA is a potential treatment option to reverse the acquired resistance of EGFR-TKIs in NSCLC.

In NSCLC-targeted therapies, autophagy is referred to as a “double-edged sword” because it promotes both cell death and survival (Li et al., 2019; Wang et al., 2021). In our experiments, we found stronger activation of autophagy in drug-resistant models both *in vivo* and *in vitro*. The antitumor effects of EGFR-TKIs were significantly enhanced when we inhibited autophagy with an autophagy inhibitor. All these results suggested that protective autophagy is one of the mechanisms of acquired EGFR-TKI resistance in NSCLC.

Further, we explored the mechanism of protective autophagy occurrence in NSCLC. Fu et al. found that when EZH2 expression was suppressed, the expression of TSC2, an mTOR signaling pathway suppressor, was upregulated, causing inhibition of the mTOR signaling pathway (Wei et al., 2015). mTOR is a major regulator of cell growth and metabolism, promoting anabolic processes and inhibiting catabolic processes, such as autophagy (Johnson and Tee, 2017). In our study, we showed that EGFR-TKIs caused significant downregulation of EZH2 and increased autophagy in NSCLC cells and tissues. siEZH2 was applied to inhibit EZH2 expression in drug-resistant cells, and we found that the changes of TSC2, p-mTOR, and autophagy were the same as those observed in drug-resistant cells treated with EGFR-TKIs alone. Altogether, we conclude that EGFR-TKIs can mediate autophagy through the EZH2/TSC2/p-mTOR signaling pathway to develop acquired drug resistance.

EZH2, with histone methyltransferase (HMTase) activity, is an important epigenetic regulator. It catalyzes the trimethylation of histone H3 lysine 27 (H3K27me3), leading to transcriptional silencing (Zhang et al., 2020). Similarly, the amino acid sequence of EZH2 protein makes it suitable for covalent modifications including phosphorylation, acetylation, O-GlcNAcylation, methylation, ubiquitination, and SUMOylation modifications (Cha et al., 2005; Riising et al., 2008; Lo et al., 2018; Li et al., 2020). In this study, we found that the acetylation level of EZH2 in drug-resistant cells was significantly lower than parental sensitive cells, suggesting that deacetylase inhibitors may be a new strategy to reverse EGFR-TKI acquired resistance in NSCLC. Studies have reported that EZH2 can be acetylated by the acetyltransferase PCAF and deacetylated by the deacetylase SIRT1, which alter the state of the PRC2 complex (Wan et al., 2015). However, no significantly change of SIRT1 was found between drug-resistant cell lines and parental sensitive cell lines when treated with EGFR-TKIs (Supplementary Figure S1E), which suggested that EZH2 deacetylation caused by EGFR-TKIs is an SIRT1-independent process. SAHA can inhibit a wide range of histone deacetylases and has been identified to have antitumor effects in lymphoma, breast cancer, and lung cancer (Alqosaibi et al., 2022). Interestingly, when drug-resistant cells were treated with SAHA alone, there were no significant changes in EZH2 expression. However, the decreasing of EZH2 and increasing of autophagy induced by EGFR-TKIs were reversed when treatment was combined with EGFR-TKIs and SAHA. These results suggests that SAHA has a potential role in overcoming acquired resistance of EGFR-TKIs in NSCLC. Furthermore, we confirmed that the combination of SAHA and EGFR-TKIs had a stronger antitumor effect than EGFR-TKIs alone in both *in vivo* and *in vitro* drug-resistant models. Nevertheless, the correlation of EZH2 deacetylation, EZH2 expression, and acquired resistance of EGFR-TKIs is still unclear and needs to be further explored.

In conclusion, our study suggests that combined treatment with SAHA and EGFR-TKIs is a potential treatment to overcome acquired resistance to EGFR-TKIs in NSCLC.

## Data Availability

The original contributions presented in the study are included in the article/Supplementary Material, further inquiries can be directed to the corresponding authors.
