# Supplementary figures and images for: Combining EGFR-TKI With SAHA Overcomes EGFR-TKI-Acquired Resistance by Reducing the Protective Autophagy in Non-Small Cell Lung Cancer
Source: Front Chem. 2022 Mar 25;10:837987. doi: 10.3389/fchem.2022.837987 (PMC8990828; doi:10.3389/fchem.2022.837987)

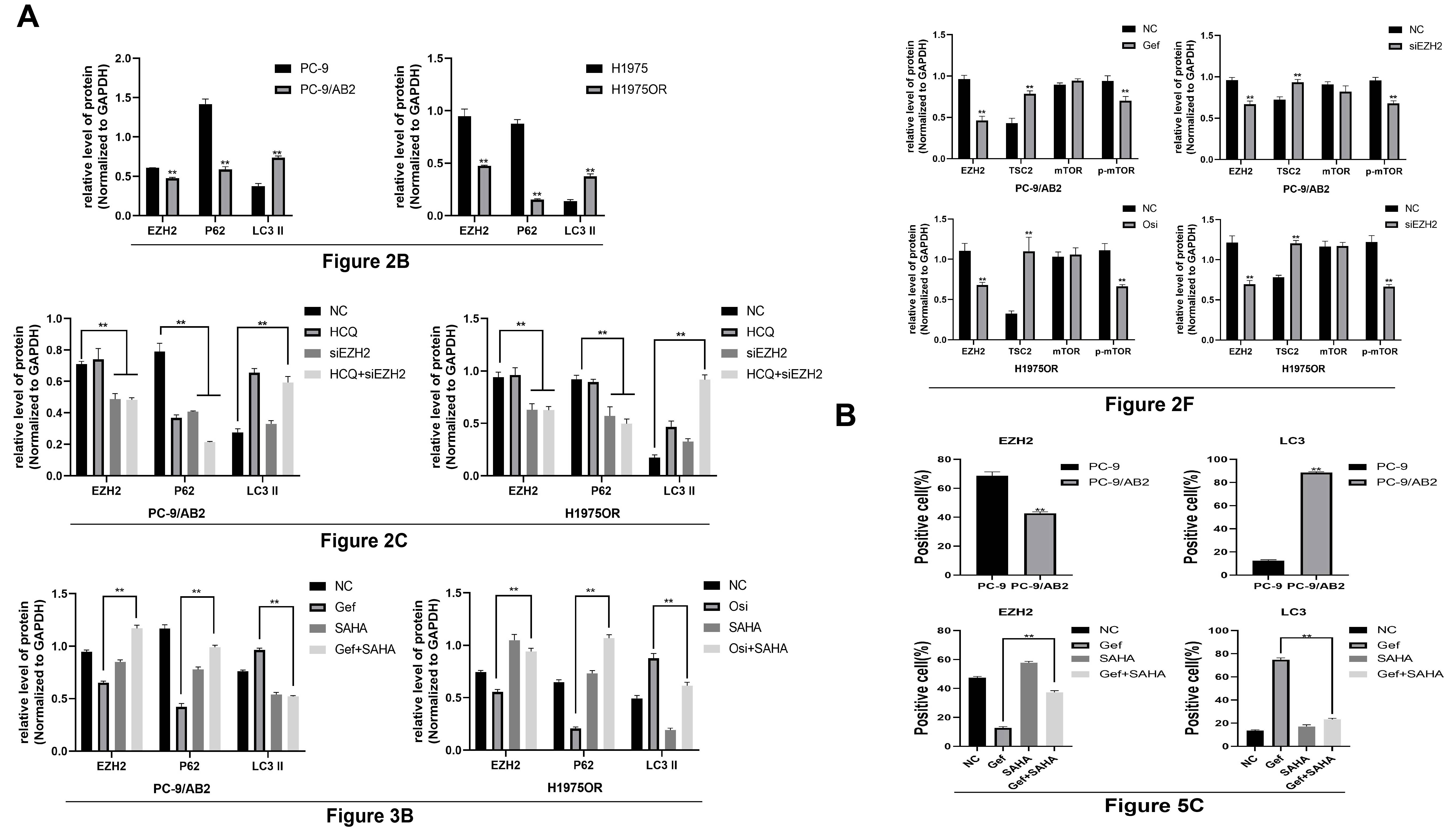

Supplement: Supplementary file 1 [file Image2.JPEG]

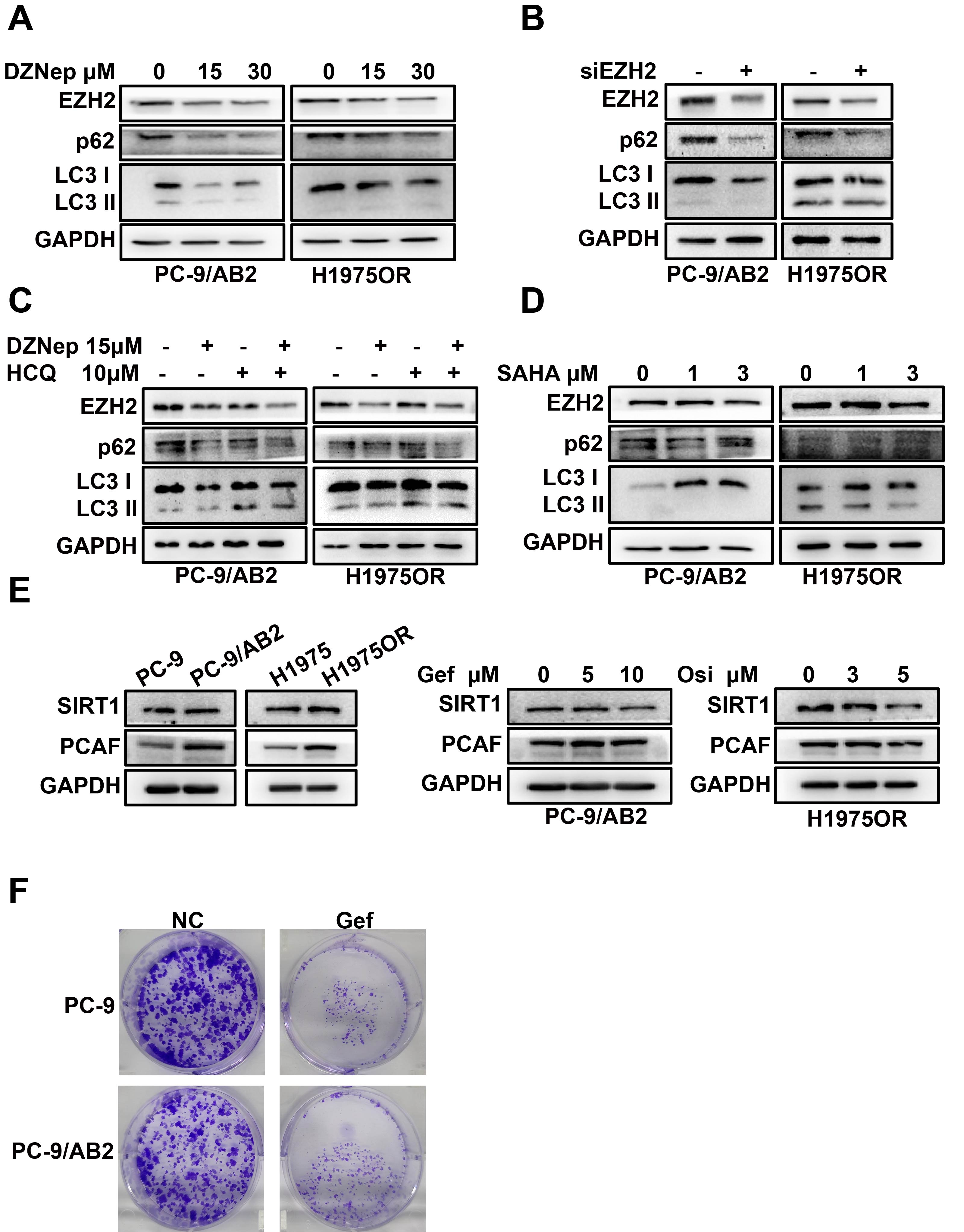

Supplement: Supplementary file 2 [file Image1.jpg]
